# S1PR3, as a Core Protein Related to Ischemic Stroke, is Involved in the Regulation of Blood–Brain Barrier Damage

**DOI:** 10.3389/fphar.2022.834948

**Published:** 2022-05-24

**Authors:** Xuehui Fan, Hongping Chen, Chen Xu, Yingju Wang, Pengqi Yin, Meng Li, Zhanbin Tang, Fangchao Jiang, Wan Wei, Jihe Song, Guozhong Li, Di Zhong

**Affiliations:** Department of Neurology, The First Affiliated Hospital of Harbin Medical University, Harbin, China

**Keywords:** ischemic stroke, S1PR3, blood–brain barrier, cerebral ischemia/reperfusion injury, WGCNA analysis

## Abstract

**Background:** Ischemic stroke is the most common stroke incident. Sphingosine-1-phosphate (S1P) receptor 3 (S1PR3) is a member of the downstream G protein-coupled receptor family of S1P. The effect of S1PR3 on ischemic stroke remains elusive.

**Methods:** We downloaded two middle cerebral artery occlusion (MCAO) microarray datasets from the Gene Expression Omnibus (GEO) database and screened differentially expressed genes (DEGs). Then, we performed a weighted gene coexpression network analysis (WGCNA) and identified the core module genes related to ischemic stroke. We constructed a protein–protein interaction (PPI) network for the core genes in which DEGs and WGCNA intersected. Finally, we discovered that S1PR3 was involved as the main member of the red proteome. Then, we explored the mechanism of S1PR3 in the mouse tMCAO model. The S1PR3-specific inhibitor CAY10444 was injected into the abdominal cavity of mice after cerebral ischemia/reperfusion (I/R) injury, and changes in the expression of blood–brain barrier-related molecules were measured using PCR, western blotting, and immunofluorescence staining.

**Results:** Both GEO datasets showed that S1PR3 was upregulated during cerebral I/R in mice. WGCNA revealed that the light yellow module had the strongest correlation with the occurrence of IS. We determined the overlap with DEGs, identified 146 core genes that are potentially related to IS, and constructed a PPI network. Finally, S1PR3 was found to be the main member of the red proteome. In the mouse cerebral I/R model, S1PR3 expression increased 24 h after ischemia. After the administration of CAY10444, brain edema and neurological deficits in mice were ameliorated. CAY10444 rescued the decreased expression of the tight junction (TJ) proteins zonula occludens 1 (ZO1) and occludin after ischemia induced by transient MCAO (tMCAO) and reduced the increase in aquaporin 4 (AQP4) levels after tMCAO, preserving the integrity of the BBB. Finally, we found that S1PR3 is involved in regulating the mitogen-activated protein kinase (MAPK) and (phosphatidylinositol-3 kinase/serine-threonine kinase) PI3K-Akt signaling pathways.

**Conclusion:** S1PR3 participates in the regulation of blood–brain barrier damage after cerebral I/R. S1PR3 is expected to be an indicator and predictor of cerebral ischemia, and drugs targeting S1PR3 may also provide new ideas for clinical medications.

## Introduction

Acute ischemic stroke (IS) accounts for >80% of all strokes (Cheng et al., 2020). The global burden of ISs is approximately fourfold higher than hemorrhagic strokes (Zhao et al., 2019). Tissue plasminogen activator (tPA) is the only Food and Drug Administration- (FDA-) approved treatment for acute IS (Chan et al., 2010). The pathogenesis of acute is highly complex and involves multiple mechanisms. The pathogenesis of cerebral I/R injury is a rapid cascade reaction that includes many linked pathways. The main links are the imbalance of intracellular calcium homeostasis, the instability of the amino acid content in brain tissue, the generation of free radicals, the inflammatory response, the activation of apoptotic genes, and energy disorders. The blood–brain barrier (BBB) is primarily formed by specialized brain endothelial cells that are interconnected by well-developed tight junctions (TJs) and provides a dynamic interface between the blood and the brain (Jin et al., 2010). BBB disruption is a critical event in the pathogenesis of acute IS; however, the molecular mechanisms involved are not completely understood.

We focused our attention on IS, downloaded two middle cerebral artery occlusion (MCAO) microarray datasets from the GEO database, screened them for differentially expressed genes (DEGs), performed weighted gene coexpression network analysis (WGCNA) analysis, and identified core genes related to IS. We used the core genes at the intersections of modular genes, DEGs, and WGCNA to construct the protein–protein interaction (PPI) network and finally determined the expression of the core gene sphingosine-1-phosphate (S1P) receptor 3 (S1PR3). S1PR3 is a member of the family of G protein-coupled receptors downstream of S1P. The metabolites of sphingolipids include ceramide, sphingomyelin, and S1P. As the active product of sphingosine, S1P is a natural lipid with biological activity. S1P is mainly produced from sphingomyelin on the cell membranes of red blood cells, platelets, and endothelial cells through a reaction catalyzed by various enzymes (O'Sullivan and Dev, 2013). After S1P is produced, it plays a role in and outside the cell. S1P exerts its regulatory effect by binding to five G protein-coupled receptors, S1PR1-S1PR5, which activate downstream signaling pathways, such as Ras/extracellular signal-regulated kinase (ERK) 1/2 and phosphatidylinositol-3 kinase/serine-threonine kinase (PI3K-Akt), and exerts a variety of biological effects (Patmanathan et al., 2017). The distribution of S1P receptors shows tissue selectivity. S1PR1, S1PR2, and S1PR3 are widely expressed in tissues. The expression of S1PR4 is limited to lymphocytes, while S1PR5 is mainly expressed in the nervous system (Sanchez and Hla, 2004).

The central nervous system is rich in S1P, and the content of SIP in the brain is higher than that in other organs, suggesting that it may be an important source of S1P in the blood. The destruction of the BBB caused by cerebral ischemia initiates the development of brain edema and the progression of brain damage (Bai et al., 2014). In this article, we used the S1PR3-specific antagonist CAY-10444, evaluated the neurological scores and brain water content of mice after cerebral I/R, and finally explored whether S1PR3 is involved in the process of cerebral ischemia/reperfusion (I/R) injury by regulating BBB injury.

## Materials and Methods

### Datasets and Data Preprocessing

**FIGURE 1:**
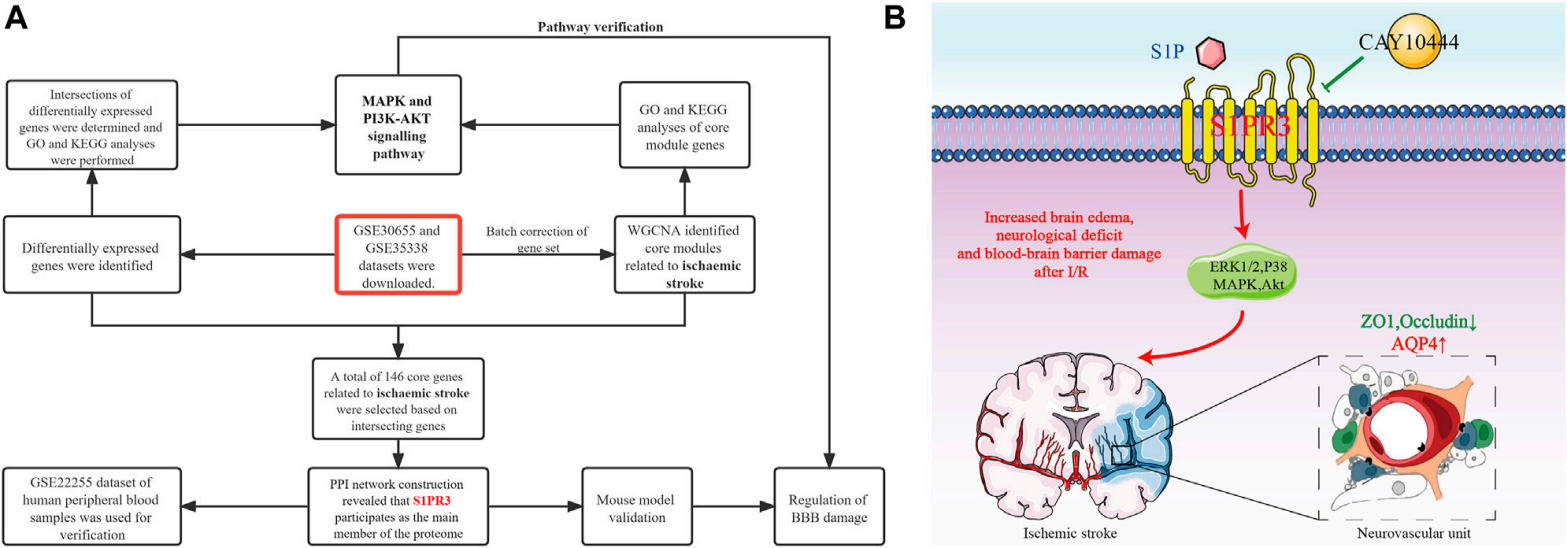
Flowchart of the study **(A)** and S1PR3 control mode diagram **(B)**.

Two microarray datasets (GSE30655 and GSE35338) based on the GPL1261 platform were downloaded from the Gene Expression Omnibus (GEO) database (http://www.ncbi.nlm.nih.gov/geo) as a screening set. The datasets were collected from the brain tissues of model mice and normal mice subjected to MCAO. Finally, three normal samples from GSE30655 and four normal samples from GSE35338 were included, including seven MCAO samples in GSE30655 and five MCAO samples in GSE35338. The GSE22255 dataset based on the GPL570 platform was downloaded for verification. This dataset was used for gene expression profiling in peripheral blood mononuclear cells (PBMCs) from 20 patients with IS and 20 healthy people. In addition, in WGCNA (Zhang and Horvath, 2005), the sva program package was used to perform background correction and normalization and calculate the expression in the GSE30655 and GSE35338 datasets. PCA shows that this analysis method better eliminates the batch effect (Supplementary Figure S1). The flowchart of the analysis is shown in Figure 1A.

### Screening of Markers

First, we screened DEGs in the screening set and used the limma package to determine the DEGs between MCAO samples and normal samples. We selected |log2 fold change (FC)|>1 and adjusted *p*-value < 0.05 as the thresholds. In WGCNA, after batch correction, all DEGs with a *p*-value < 0.05 in normal samples and MCAO samples were used as input. The clustering of each sample was good, and no samples were excluded. Subsequently, the topological calculation was performed with a soft threshold value ranging from 1 to 20, and the optimal soft threshold value was determined to be 14. According to the soft threshold, the relationship matrix was converted into an adjacent matrix and then converted into a topological overlap matrix (TOM), and an average link hierarchical clustering analysis was performed. The related modules were classified according to the TOM, and the number of genes in each module was not less than 50. The shear height of the gene modules in this study was 0.7, and similar modules were merged. In addition, the gene significance (GS) and module member (MM) in each module were calculated to draw scatter plots. Finally, the Pearson method was used to calculate the correlation between the combined module and MCAO.

### Enrichment Analysis

Gene Ontology (GO) enrichment analysis is a commonly used bioinformatics method for searching for comprehensive information in large-scale genetic data, including biological processes (BPs), molecular functions (MFs), and cellular components (CCs). (Subramanian et al., 2005). In addition, the Kyoto Encyclopedia of Genes and Genomes (KEGG) pathway enrichment analysis is widely used to understand biological mechanisms and functions (Kanehisa et al., 2019). The GO and KEGG analyses were visualized using the GOplot package. The org.Mm.eg.db package was used to annotate the corresponding entryID of the gene, and then enrichment analysis and visualization were performed using clusterProfiler, enrichplot, and ggplot2 packages. In this study, we conducted two gene enrichment analyses, and the thresholds used were a *p*-value < 0.05 and *q*-value > 0.05.

### PPI Network

The Metascape database (http://metascape.org) was used to construct the PPI network of these specific genes, and the MCODE method was used to identify the closely related proteome in the PPI network. Cytoscape software was used to visualize the network.

### Animal Studies

For this experiment, C57BL/6 male mice were selected. The mice were SPF grade and weighed approximately 20–25 g. All experimental mice were purchased from Liaoning Changsheng Biotechnology Co., Ltd. All experimental animals were managed and used strictly in accordance with the experimental animal management guidelines of the First Affiliated Hospital of Harbin Medical University, as recommended by the US National Institutes of Health. During the experiment, the mice were housed in an environment with a humidity of 50%–60% and a temperature of 23°C–25°C, the natural circadian rhythm was simulated with a 12 h/12 h alternating light mode, and the mice were able to eat and drink water freely.

### Construction of the Transient MCAO

Cerebral ischemia was established by generating the tMCAO model using a modified intraluminal technique (Liu et al., 2010). The mice were anesthetized with 3% pentobarbital sodium, their heads were shaved and disinfected, and the anal temperature probe was inserted to keep the body temperature at 37 ± 0.5°C. The skin of the neck was cut open to separate the common carotid artery, internal carotid artery, and external carotid artery. An incision was made on the right common carotid artery, and a 0.21 mm thread was inserted into the internal carotid artery through the common carotid artery until the middle cerebral artery, and the depth reached about 9± 1 mm at the bifurcation of the internal and external carotid arteries. If there was any resistance, the thread was stopped. One hour after the ischemia, the threaded plug was removed, the skin was sutured, and the mouse was placed on a heating pad. After being awake, the mouse was placed in a constant temperature rearing box, and then the mice were allowed to recover for 6 h, 12 h, 1 d, 3 d, and 5 d. The animals in the sham group were subjected to the same operation process except that the MCA was not occluded. CAY-10444 was purchased from the Cayman Company and was injected intraperitoneally into mice at 0.5 mg/kg during reperfusion (Gaire et al., 2018). CAY-10444 was solubilized in dimethylsulfoxide (DMSO, less than 2%). Mice in the V + tMCAO group were intraperitoneally injected with vehicle (DMSO, less than 2%) after ischemia and reperfusion. Mice were randomly divided into the following groups: 1) sham group; 2) tMCAO group; 3) CAY-10444 + tMCAO group; and 4) V + tMCAO group.

### Assessment of Neurological Deficits

An inspector unknowingly scored mice for neurological deficits. The deficits were scored as follows: 0, no deficits; 1, forelimb weakness and torso turning to the ipsilateral side when held by the tail; 2, unable to extend the opposite forepaw completely; 3, turning to the paralyzed side; 4, dumping to the opposite side; and 5, unable to walk spontaneously and loss of consciousness.

### Brain Water Content Measurement

The wet and dry weight method is used (Hatashita et al., 1988). A tissue was removed from the right side of the brain and placed in a Petri dish with a qualitative filter moistening with normal saline to prevent evaporation. The time from brain extraction to weighing was controlled within 5 min. Then, it is baked at 100°C in an electric thermostat until constant weight. The brain water content (%) = (wet weight − dry weight)/wet weight ×100%.

### Evans Blue Staining and Content Determination

After the successful production of the mouse tMCAO model, each group was intraperitoneally injected with drugs and solvents, followed by an immediate intraperitoneal injection of 2% Evans blue solution (4 ml/kg). After 24 h, the heart was perfused with normal saline; the brain tissue was taken out quickly and weighed; and 50% trichloroacetic acid was added, homogenized by a homogenizer, at 4°C, and centrifuged at 3000 rpm/min for 20 min. The absorbance of the supernatant was measured by a spectrophotometer at 620 nm. Draw the standard curve according to the instructions, dilute Evans blue with normal saline, take appropriate amount to add trichloroacetic acid solution, and then dilute, spectrophotometer to measure the wavelength, and finally calculate the content of Evans blue according to the absorbance.

### Double-Immunofluorescence Staining

Brain sections were fixed with 4% paraformaldehyde. After permeabilization and blocking, the samples were incubated at 4°C overnight with primary antibodies specifically raised against the following proteins: CD31 (GeneTex, GTX20218, 1:100), zonula occludens 1 (ZO1) (Abmart, TA5145, 1:100), occludin (GeneTex, GTX 114949, 1:100), aquaporin 4 (AQP4) (Proteintech, 16473-1-AP, 1:100), and matrix metalloproteinase-9 (MMP9) (GeneTex, GTX100458, 1:50). Subsequently, the samples were incubated with the appropriate fluorophore-conjugated secondary antibodies (BOSTER, BA1089, and BA1105, 1:100) for 1 h at room temperature in the dark. DAPI (Abcam, ab104139) was used to stain cell nuclei. Images were captured using a fluorescence microscope (Nikon, Y-TV55, JAPAN). We used ImageJ to analyze the immunofluorescence area, and the fluorescence images were imported into the software. Ensure the image is eight bits, and convert to grayscale to best visualize all positive staining. Then, adjust brightness and contrast, go to “Image” and select “Adjust” and “Threshold.” Adjust the upper and lower pulleys, select the positive signal area, and click “Analyze” and “Measure” to obtain statistical results. The immunofluorescence image area we selected is the peri-ischemic regions of the ischemic hemisphere. The number of mice in each group was four, and each group had at least four independent sections. The experiment was double-blind, and the group members were not the same as the experimenter who read images and performed statistics.

### Quantitative Real-Time PCR

TRIzol reagent (TaKaRa, Japan) was used for PCR, and the fasting one-step method was used to remove genomic DNA. The cDNA first-strand synthesis premixes the reagent to synthesize first-strand cDNAs (TIANGEN, China). Finally, quantitative PCR was performed using a PCR machine (Bio-Rad, Singapore) with the SYBR green kit (TIANGEN, China), and the relative gene expression level was normalized to GAPDH. The primer sequences were as follows:

Occludin: F: TTG​AAA​GTC​CAC​CTC​CAC​CTC​CTT​ACA​GA, R: CCG​GAT​AAA​AAG​AGT​ACG​CTG​G;

ZO1: F: GCC​GCT​AAG​AGC​ACA​GCA​A, R: GCC​CTC​CTT​TTA​ACA​CAT​CAG​A;

MMP9: F: GCA​GAG​GCA​TAC​TTG​TAC​CG, R: TGA​TGT​TAT​GAT​GGT​CCC​ACT​TG; AQP4: F: CTT​TCT​GGA​AGG​CAG​TCT​CAG, R: CCA​CAC​CGA​GCA​AAA​CAA​AGA​T; and GAPDH: F: GGT​GTG​AAC​CAT​GAC​AAG​TAT​GA, R: GAG​TCC​TTC​CAC​GAT​ACC​AAA​G.

### Western Blot Analysis

Brain tissue from the right hemisphere was obtained, and proteins were extracted on the first day after ischemia and reperfusion. A BCA protein concentration determination kit (Beyotime Biotechnology, China) was used to determine the protein concentration. In addition, 30 µg of protein from each group were loaded onto an 8% or 10% SDS-PAGE gel. After electrophoresis, the brain proteins were transferred to polyvinylidene fluoride (PVDF) membranes, blocked with 5% skim milk at room temperature for 1 h, and then incubated with the primary antibody overnight in a 4°C refrigerator. After incubation with goat anti-mouse and anti-rabbit (Abmart, M21003, 1:2,000) secondary antibodies for 45 min at room temperature, membranes were washed with TBST and then incubated with enhanced chemiluminescence (ECL) reagent (Biosharp, BL520A, China) for detection. Primary antibodies included anti-S1PR3 (1:2,000, ab108370, Abcam, United States), anti-AQP4 (1:1,000, 16473-1-AP, Proteintech, United States), anti-MMP9 (1:1,000, 16375-1-AP, Proteintech, United States) anti-ZO1 (1:1,000, 21773-1-AP, Proteintech, United States), anti-Occludin (1:1,000, Abcam, United States), anti-ERK1/2 (1:2000, AF0155, Affinity, United States), anti-phospho-ERK1/2 (1:1,000, AF1015, Affinity, United States), anti-AKT1/2/3 (1:1,000, AF6261, Affinity, United States), anti-phospho-AKT1/2/3 (1:1,000, AF0016, Affinity, United States), anti-p38MAPK (1:1,000, AF6456, Affinity, United States), and anti-phospho-p38MAPK (1:1,000, AF4001, Affinity, United States). β-Tubulin (1:1 000, 10094-1-AP, Proteintech, United States) was used for internal comparison. ImageJ software was used to quantitatively analyze the grey values of all protein bands.

### Statistical Analysis

GraphPad Prism software 8.0 was used for the statistical analysis. The data are presented as the means ± SEM. Differences between groups were analyzed using one-way ANOVA followed by the Tukey post hoc test. *p* < 0.05 was regarded as statistically significant.

## Results

### Expression and Identification of Differentially Expressed Genes in tMCAO Mice

**FIGURE 2:**
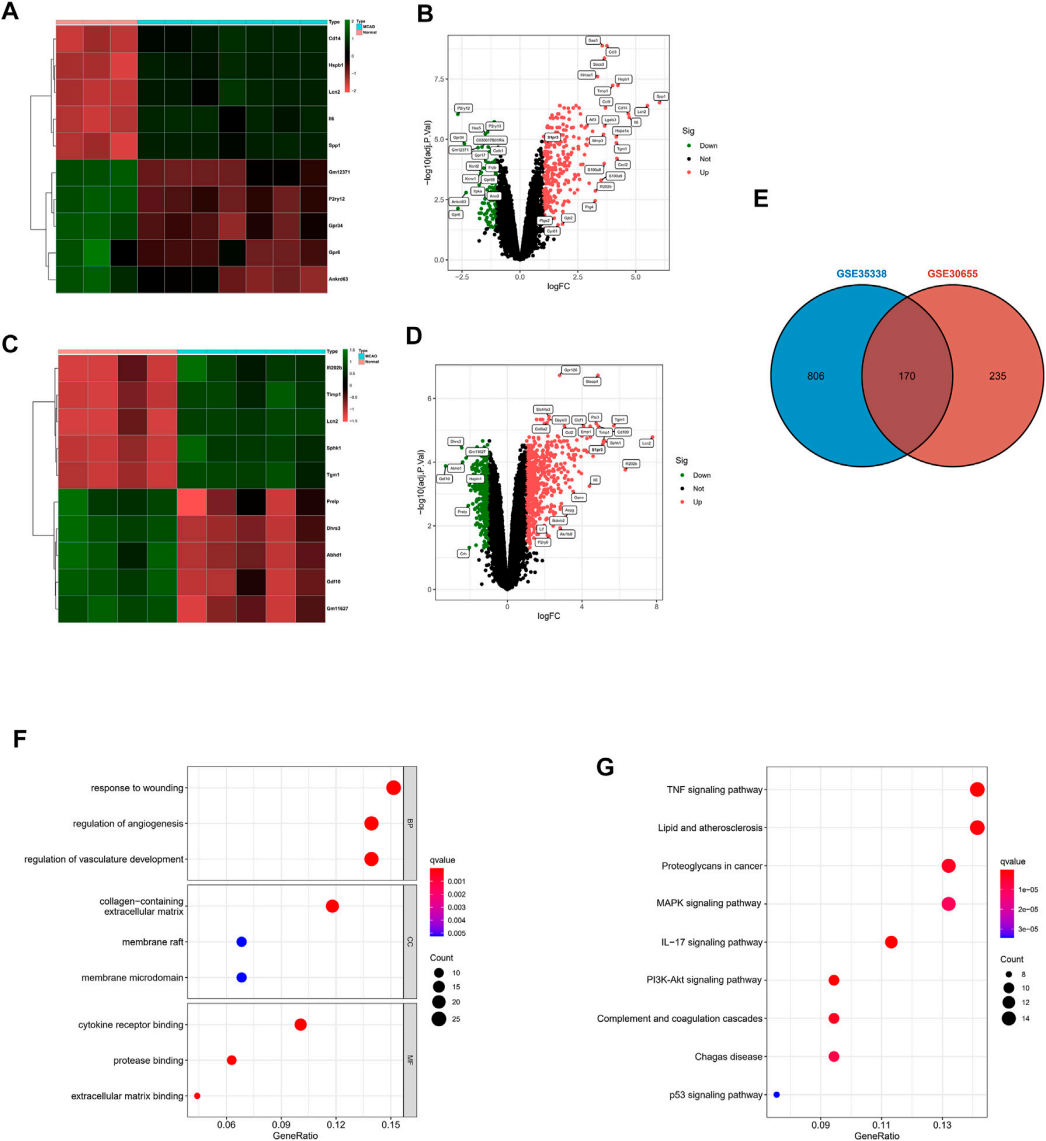
The expression and identification of differentially expressed genes in the tMCAO model. **(A,B)** Heatmap and volcano plot of differentially expressed genes in GSE30655. **(C,D)** Heatmap and volcano plot of differentially expressed genes in GSE35338. **(E)** Venn diagram of the intersecting the DEGs from the two GEO datasets. The bar plot shows the GO functional enrichment analysis **(F)** and KEGG pathway analysis **(G)** of DEGs. The color indicates the importance of the *p*-value.

First, we identified 405 DEGs in GSE30655. The heatmap shows the top 10 differentially expressed genes based on logFC (Figure 2A). The volcano plot shows 301 upregulated genes and 104 downregulated genes (Figure 2B). S1PR3 was upregulated in tMCAO mice. A total of 976 DEGs were identified in GSE35338. The heatmap also shows the top 10 differentially expressed genes based on logFC (Figure 2C). The volcano plot shows 631 upregulated genes and 345 downregulated genes (Figure 2D). S1PR3 was also upregulated in tMCAO mice. Ultimately, 170 common DEGs were obtained from the two datasets (Figure 2E). GO analysis was performed to explore the 170 common DEGs and explain their possible biological mechanisms, such as response to wounding, regulation of angiogenesis, and regulation of vasculature development (Figure 2F). At the same time, the KEGG enrichment analysis also suggested at the tumor necrosis factor (TNF) signaling pathway, MAPK signaling pathway, PI3K-Akt signaling pathway, and other pathways played an important role in the occurrence and development of IS (Figure 2G).

### Construction of WGCNA and Screening of Core Modules Related to IS

**FIGURE 3:**
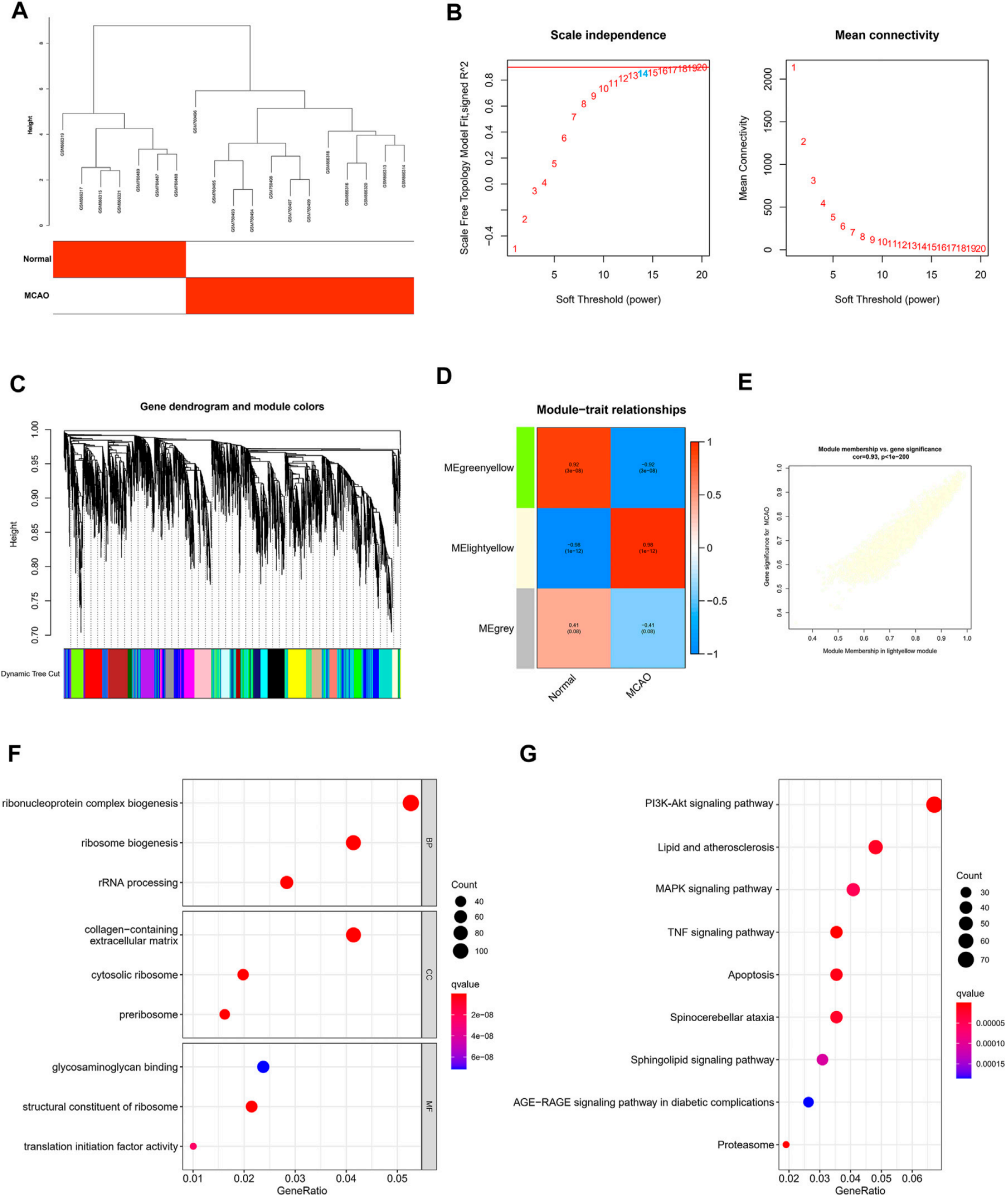
Construction of WGCNA and screening of core modules related to ischemic stroke. **(A)** Sample clustering was performed based on the information from the two datasets. All differentially expressed genes with *p* < 0.05 were used for WGCNA **(B)**. Analyses of the scale-free ft index and the mean connectivity at various soft-thresholding powers. **(C)** Gene tree diagram. The colored line below the tree diagram shows the modules determined using dynamic tree cutting. **(D)** Heatmap of the relevance between the characteristic genes of the module and the pyrolysis pattern. **(E)** Gene significance (GS) scatter plot of MCAO and module members (MMs) in the light yellow module. The bar plot displays the GO functional enrichment analysis **(F)** and KEGG pathway analysis **(G)** of the core module genes. The color indicates the importance of the *p*-value.

The WGCNA was performed to determine the associations of clinical information with key genes. After the two gene sets described above were corrected in batches, the difference analysis was only satisfied for the expression of 4,118 genes with an adjusted *p*-value < 0.05, which were included as the input matrix. The clustering of each sample was better (Figure 3A). Subsequently, the topological calculation was performed with a soft threshold ranging from 1 to 20, and the optimal soft threshold was determined to be 14 (Figure 3B). According to the soft threshold, the relationship matrix was converted into an adjacent matrix and then converted into a topological overlap matrix (TOM), and the average link hierarchical clustering analysis was performed. The related modules were classified according to the TOM. The number of genes in each module was not less than 50, and similar gene modules were merged (Figure 3C). Finally, three modules were identified. In addition, we calculated the correlation between genes and clinical traits in the module and found that the light yellow module had the strongest correlation with the occurrence of IS. Therefore, this module was used as the core module (Figure 3D). In addition, the GS and MM of 2,442 genes in the light yellow module were calculated, and a correlation scatter diagram was drawn (Figure 3E). We found that the GS and MM genes in the core module had a strong correlation, which also verified our hypothesis from another perspective. GO analysis was conducted to explore the possible biological mechanisms of 2,442 genes in the core module and revealed roles for pathways such as ribonucleoprotein complex biogenesis and ribosome biogenesis (Figure 3F). At the same time, the KEGG enrichment analysis also suggested at the TNF signaling pathway, lipid and atherosclerosis, MAPK signaling pathway, IL-17 signaling pathway, PI3K-Akt signaling pathway, and other pathways played an important role in the occurrence and development of IS (Figure 3G).

### Construction of the PPI Network of Core Genes Involved in IS and Verification Using Human Blood Samples

**FIGURE 4:**
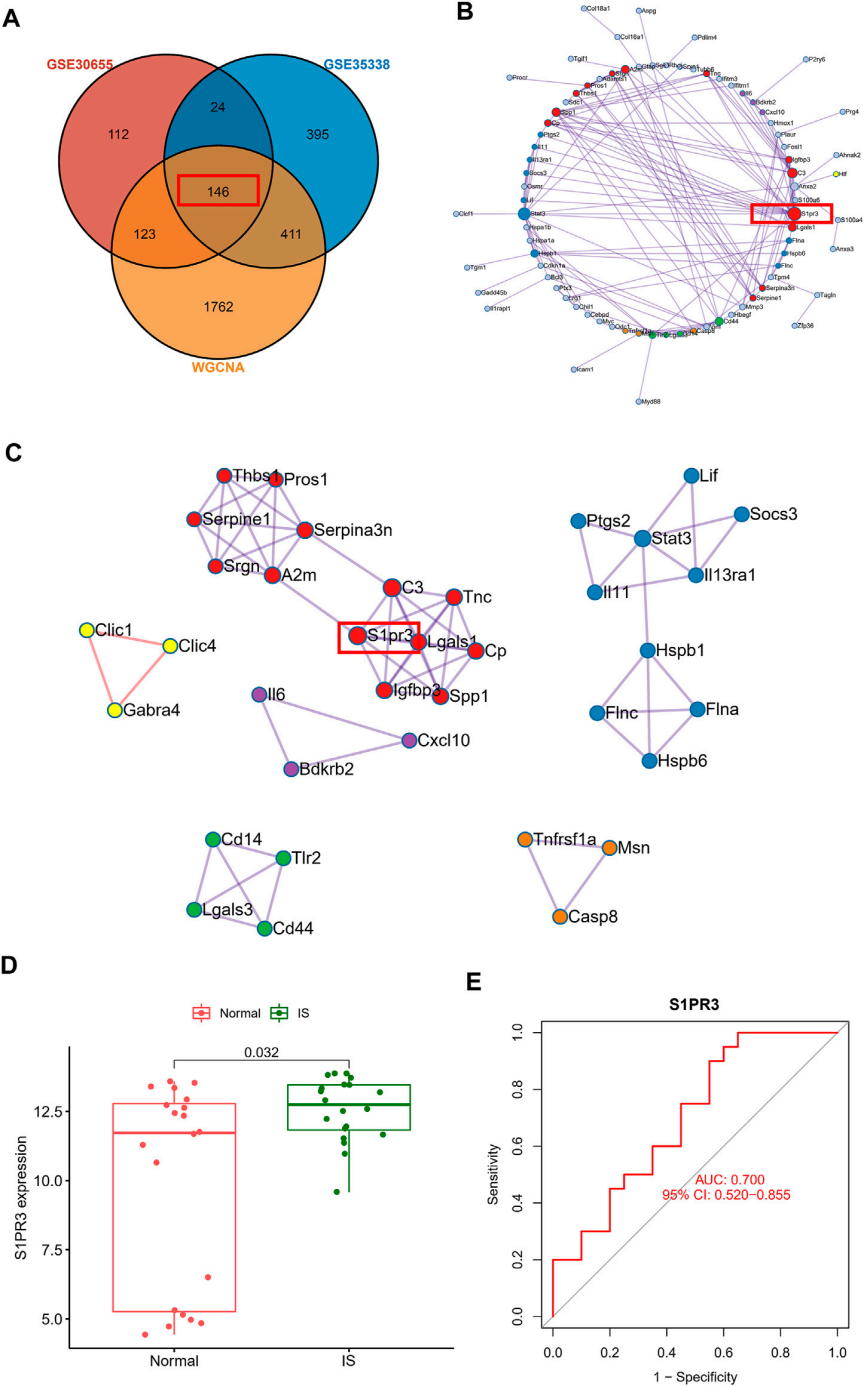
Construction of the PPI network of core genes related to ischemic stroke and verification using human blood samples. **(A)** Venn plot showing the intersections of DEGs and selected genes from WGCNA. **(B)** PPI network analysis shows the potential interrelationships of 146 genes. **(C)** Visualization of six closely related proteome MCODE modules in the PPI network. **(D)** Peripheral blood sequencing to verify the expression of S1PR3 in patients with IS. **(E)** ROC curve of S1PR3.

After intersecting the DEGs identified in the two gene sets described above and the genes in the WGCNA core module, we finally identified 146 core genes that are potentially related to IS (Figure 4A). The PPI network analysis revealed the potential interrelationships of these 146 genes (Figure 4B). The MCODE method was used to identify six closely related proteomes in the PPI network. We found that S1PR3 participates as a major member of the red proteome. We verified the clinical application value of S1PR3 in the GSE22255 peripheral blood sequencing collection and found that S1PR3 was significantly upregulated in patients with IS (Figure 4D) and was a good IS indicator (Figure 4E).

### S1PR3 Expression Is Upregulated in the Mouse Brain After tMCAO

**FIGURE 5:**
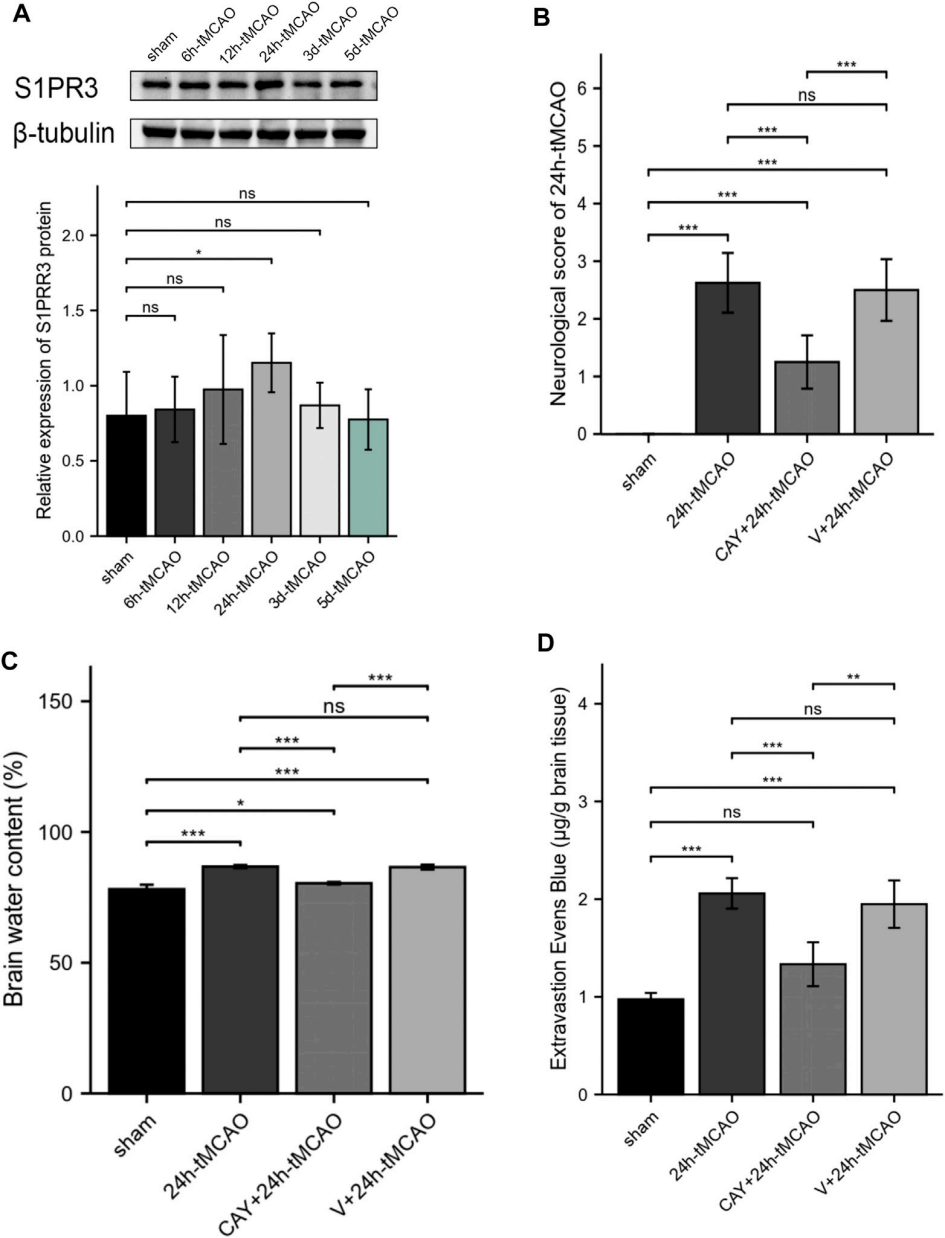
S1PR3 expression detected at different time points in tMCAO mice. Inhibition of S1PR3 ameliorates brain edema and neurological deficits in tMCAO mice. **(A)** Western blotting was used to detect the SIRT3 protein level (*n* = 4). **(B)** Neurological deficit scores of mice recorded 24 h after tMCAO (*n* = 8). **(C)** The water content of the right cerebral hemisphere of mice in each group was measured using the wet weight method and dry weight method (*n* = 4). **(D)** EB dye (2%; 4 ml/kg of body weight) was injected intraperitoneally immediately after tMCAO, and EB leakage in the brains was analyzed after 24 h of injection (*n* = 4). Data are presented as the means ± SEM. *p*-values were determined using ANOVA followed by the Tukey post hoc test. *p < 0.05; **p < 0.01; ***p < 0.001; and N.S. not significant compared to the sham group.

The expression of S1PR3 in the right hemisphere was detected at different time points after tMCAO to study the possible relevance of S1PR3 in cerebral I/R injury. Western blot experiments showed increased expression of the S1PR3 protein over time. We identified an increasing trend (*p* < 0.05) 24 h after tMCAO (Figure 5A), and its expression began to decrease on the third day.

### Inhibition of S1PR3 Ameliorates Neurological Deficits, Brain Edema, and the Permeability of BBB in Mice After tMCAO

We tested the neurological deficit scores. The neurological function scores are shown in Figure 5B. Compared with the 24 h tMCAO group, the neurological function score of the CAY-10444 administration group was significantly reduced (*p* < 0.001). We also injected mice with a specific inhibitor, CAY-10444, into the peritoneum during reperfusion. The brain water content in mice was measured to evaluate cerebral edema induced by cerebral I/R injury. Compared with the sham group, the brain water content was significantly increased after tMCAO (*p* < 0.001) (Figure 5C). The brain water content in mice from the tMCAO group treated with CAY-10444 was significantly reduced (*p* < 0.001). Compared with the V+24 h tMCAO group, the brains of mice treated with CAY-10444 also showed less water accumulation in the cells (*p* < 0.001). The BBB infiltration was assessed by the detection of Evans blue exudation. Compared with the sham group, the exudation of EB was significantly increased after tMCAO (*p* < 0.001) but decreased after CAY10444 (*p* < 0.001) (Figure 5D). It suggests that S1PR3 inhibitors improved the permeability of BBB.

### CAY-10444 Increased the Expression of Occludin and ZO1 After tMCAO in Mice

**FIGURE 6:**
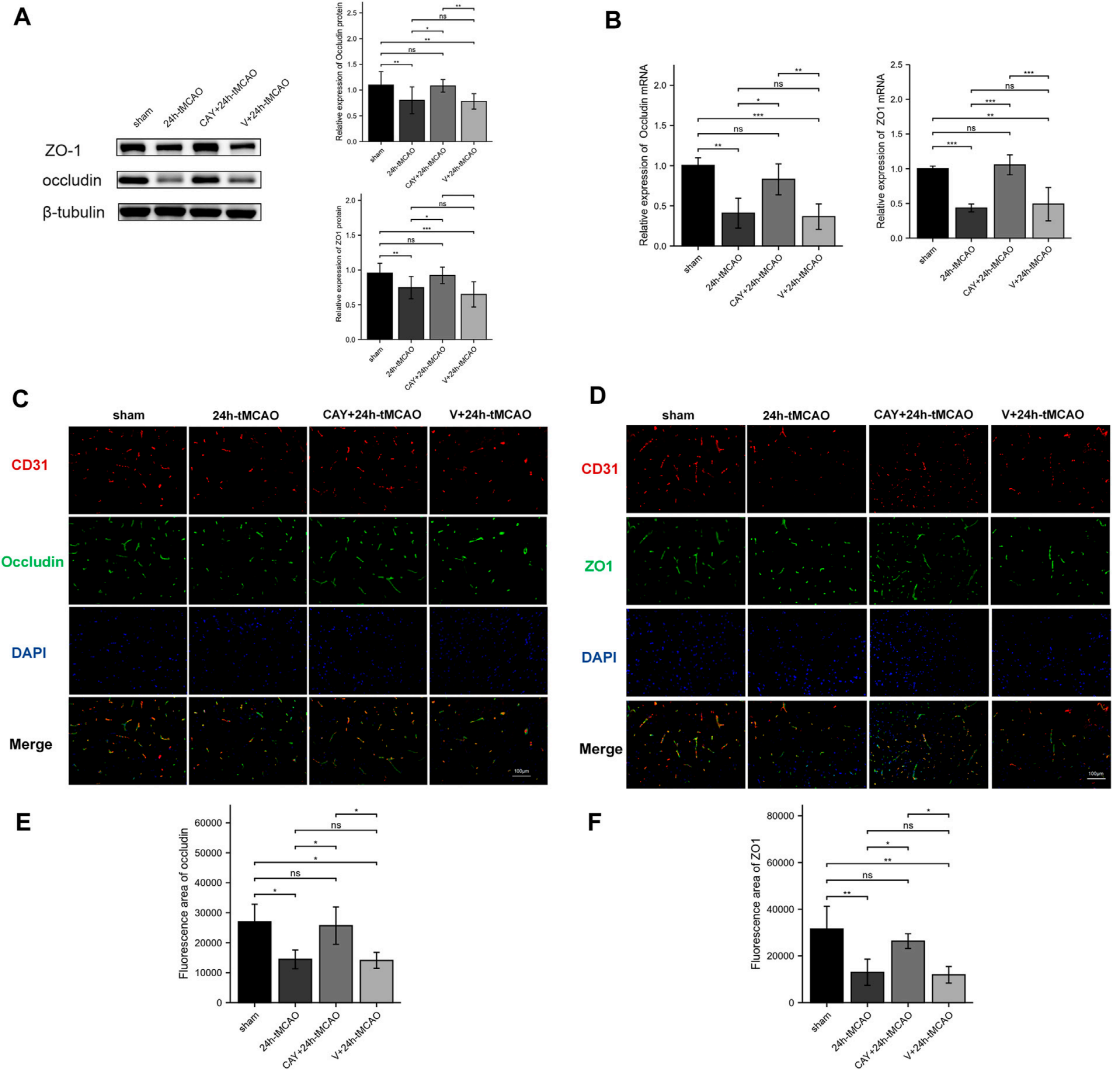
Inhibition of S1PR3 increases the expression of occludin and ZO1 in the brain tissue of mice after tMCAO. The protein **(A)** and mRNA **(B)** levels of occludin and ZO1 in the sham group, 24 h tMCAO group, CAY-10444 + 24 h tMCAO group, and V+24 h tMCAO group (*n* = 4). **(C,D)** Representative images of double-immunofluorescence staining showing the colocalization of DAPI (blue)/occludin (green) **(C)** and ZO1 (green) **(D)**/CD31 (red) (*n* = 4). **(E,F)** Immunofluorescence statistics of ZO1 and occludin (*n* = 4). Data are presented as the means ± SEM. *p*-values were determined using ANOVA followed by the Tukey post hoc test. *p < 0.05; **p < 0.01; ***p < 0.001; and N.S. not significant compared to the sham group. Scale bar = 100 µm.

The BBB is mainly composed of tight junctions (TJs) formed by protein–protein interactions between endothelial cells, such as ZO1 and occludin, which play a key role in maintaining the integrity of the BBB. Therefore, we detected the expression of these TJ proteins using Western blot and immunofluorescence analyses. On the first day after tMCAO, the expression of ZO1 and occludin decreased significantly (*p* < 0.01), indicating that the integrity of the BBB was damaged after IS. However, the administration of S1PR3 inhibitors rescued the expression of these TJ proteins after tMCAO (*p* < 0.05) (Figure 6A). Similarly, we examined the ZO1 and occludin mRNA levels. Compared with the sham group, the 24 h tMCAO group exhibited reduced expression of the ZO1 and occludin mRNAs (*p* < 0.01 and *p* < 0.001, respectively). At the same time, S1PR3 inhibition upregulated the level of the ZO1 and occludin transcript (*p* < 0.05) (Figure 6B). The results of immunofluorescence staining showed that the colocalization of TJ proteins and endothelial marker CD31 was significantly reduced after tMCAO in the peri-ischemic regions (occludin: *p* < 0.05, and ZO1: *p* < 0.01, respectively) (Figures 6E,F). However, after tMCAO, the colocalization in the CAY +24 h tMCAO group was significantly stronger than that in the 24 h tMCAO group (*p* < 0.05) (Figures 6E,F), which was consistent with the Western blot results (Figure 6A). Based on these results, S1PR3 inhibition preserves the integrity of the BBB after tMCAO.

### CAY-10444 Reduced AQP4 Expression in Mice After tMCAO

**FIGURE 7:**
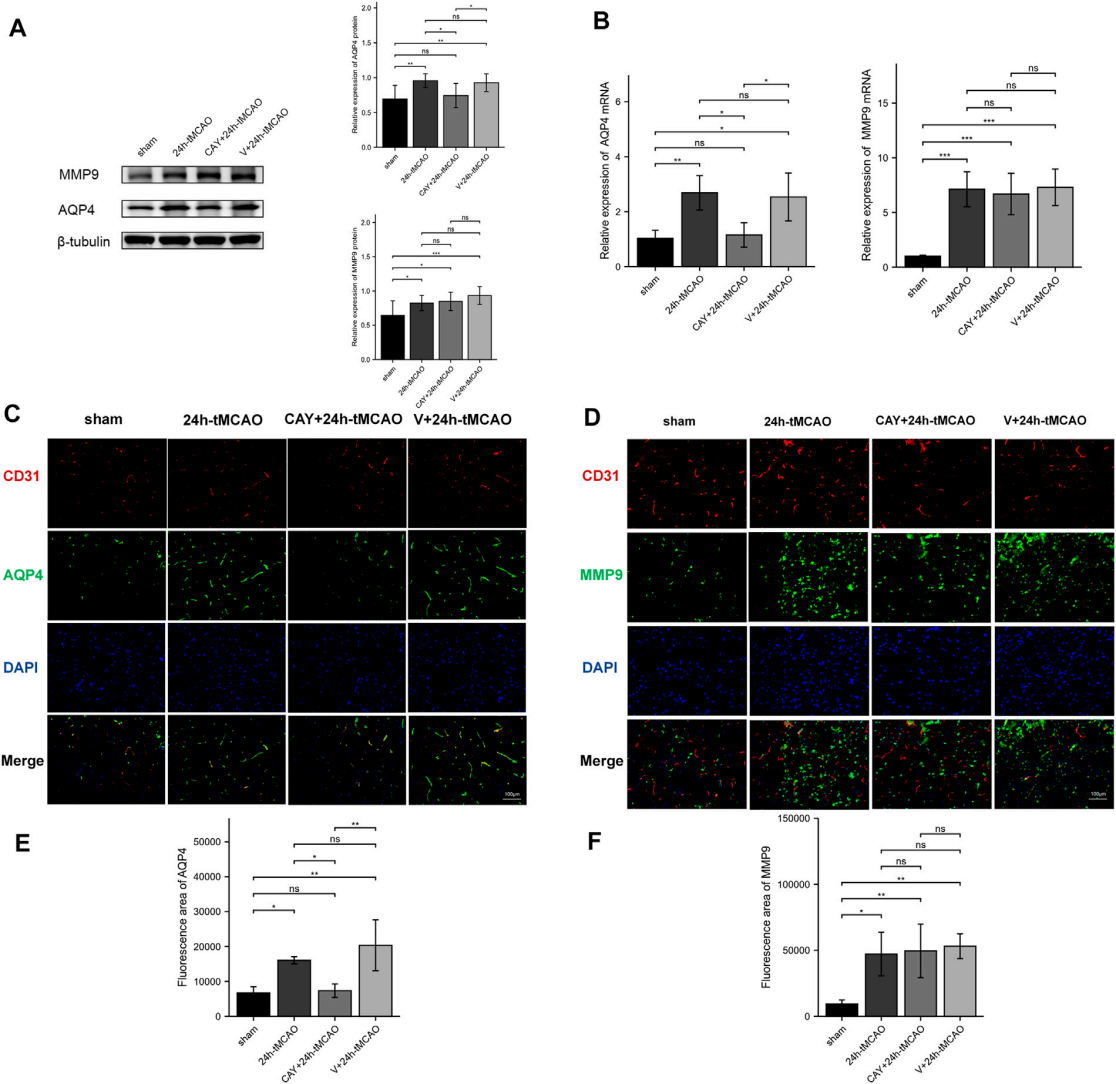
S1PR3 inhibition reduces the expression of AQP4 in the mouse brain tissue after tMCAO. Protein **(A)** and mRNA **(B)** levels of AQP4 and MMP9 in the sham group, 24 h tMCAO group, CAY-10444 + 24 h tMCAO group, and V+24 h tMCAO group (*n* = 4). **(C,D)** Representative images of double-immunofluorescence staining (n = 4) showing the colocalization of DAPI (blue)/AQP4 (green) **(C)** and MMP9 (green) **(D)**/CD31 (red). **(E,F)** Immunofluorescence statistics of AQP4 and MMP9 (*n* = 4). Data are presented as the means ± SEM. *p*-values were determined using ANOVA followed by the Tukey post hoc test. *p < 0.05; **p < 0.01; ***p < 0.001; and N.S. not significant compared to the sham group. Scale bar = 100 µm.

AQP4 and MMP9 are related to the proteolysis of the BBB after brain injury. The expression of AQP4 and MMP9 was evaluated using Western blot. Compared with the sham group, AQP4 and MMP9 levels were increased after tMCAO (*p* < 0.01 and *p* < 0.05, respectively) (Figure 7A). Then, the study explored the effects of S1PR3 inhibitors on the levels of AQP4 and MMP9 after tMCAO. Compared with the 24 h tMCAO group and the V+24 h tMCAO group (*p* < 0.05 and *p* < 0.05, respectively), CAY-10444 administration decreased the expression of AQP4 (Figure 7A). Similarly, we explored the AQP4 mRNA level and observed increased expression in the 24 h tMCAO group compared with the sham group (*p* < 0.05). At the same time, the inhibition of S1PR3 downregulated the level of the AQP4 transcript (*p* < 0.05) (Figure 7B). After treatment with CAY-10444, no significant difference in the level of MMP9 protein was observed compared with the 24 h tMCAO group and the V+24 h tMCAO group (*p* = 0.979 and *p* = 0.519, respectively). At the transcript level, the 24 h tMCAO group exhibited increased expression of the MMP9 mRNA compared with the sham group (*p* < 0.001), but inhibition of S1PR3 did not affect the level of the MMP9 transcript (*p* = 0.976) (Figure 7B). Under physiological conditions, AQP4 in mouse brain tissue is expressed in a polar form on the foot processes of perivascular astrocytes. Mice were subjected to double-immunofluorescence for AQP4 and the vascular marker CD31 to observe the changes in the polarity and distribution of AQP4 around the cerebral ischemic focus of mice after tMCAO. As shown in Figure 7C, AQP4 was coexpressed with CD31 in the brain tissues of mice in the sham group, and fluorescence staining was distributed along the blood vessels. After tMCAO for 24 h, the distribution of AQP4 around the blood vessel expanded and was distributed in the extravascular area (*p* < 0.05) (Figure 7E). After CAY-10444 was administered, immunofluorescence staining for AQP4 in the periphery of the blood vessel decreased, indicating that S1PR3 was involved in the upregulation and redistribution of AQP4 (*p* < 0.05) (Figure 7E). Immunofluorescence staining for MMP9 was lower in the sham group and distributed around the blood vessels (Figure 7D). Compared with the sham group, immunofluorescence staining for MMP9 around the cerebral ischemic focus of mice in the 24 h tMCAO group increased significantly but decreased slightly in the CAY+24 h tMCAO group (*p* < 0.05) (Figure 7F), although the levels were still greater than those in the sham group (*p* < 0.01) (Figure 7F).

### S1PR3 May Regulate BBB Damage by Activating PI3K/Akt, p38MAPK, and ERK1/2

**FIGURE 8:**
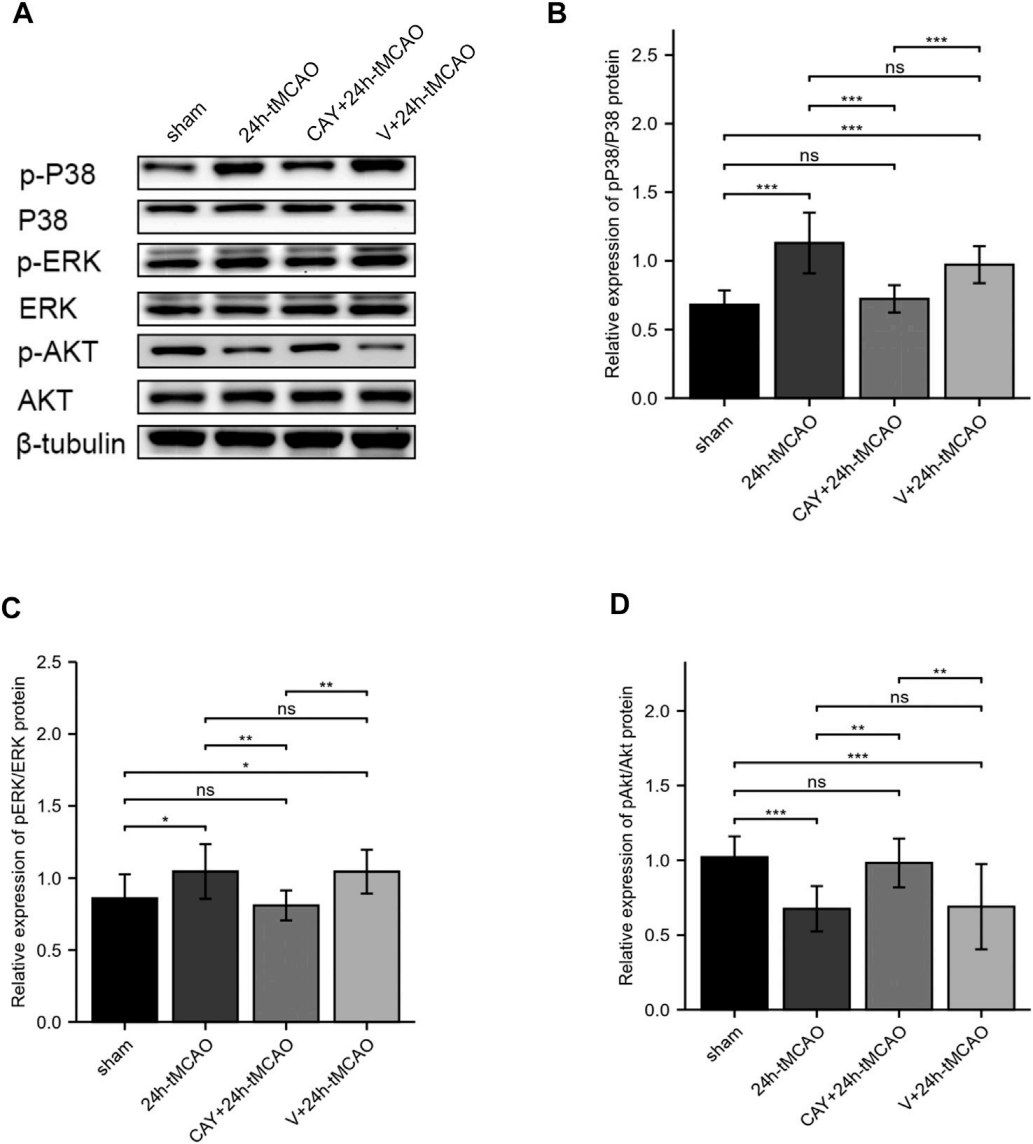
S1PR3 inhibition changes the levels of Akt, p38, and ERK1/2 phosphorylation. **(A)** Western blots showing the levels of phosphorylated p38, ERK1/2, and Akt in the sham, 24 h tMCAO, CAY-10444 + 24 h tMCAO, and V+24 h tMCAO groups. **(B–D)** Phosphorylated p38, ERK1/2, and Akt protein expression levels. Data are presented as the means ± SEM. *p*-values were determined using ANOVA followed by the Tukey post hoc test. *p < 0.05; **p < 0.01; ***p < 0.001; and N.S. not significant compared to the sham group.

In a previous KEGG analysis, we found that the MAPK signaling pathway and the PI3K-Akt signaling pathway may be important pathways regulating S1PR3 in cerebral ischemia injury. We evaluated the level of its phosphorylation form using western blotting. In the ischemic brain, p38 MAPKs and ERK1/2 were significantly activated (*p* < 0.001 and *p* < 0.05, respectively). When the S1PR3 activity was inhibited by CAY-10444, the increase in the phosphorylation of ERK1/2 and p38 MAPKs was reduced (*p* < 0.001 and *p* < 0.01, respectively) (Figures 8A–C). In contrast, Akt phosphorylation decreased after tMCAO (*p* < 0.001), and this decrease was reversed by CAY-10444 administration (*p* < 0.01) (Figures 8A,D). According to previous studies, the expression of TJ proteins is regulated by PI3K/Akt, p38MAPK, ERK1/2, TLR4, and other signal transduction pathways (Liu et al., 2014; Wang et al., 2014; Hue et al., 2015). AQP4 expression is also negatively regulated by blocking ERK1/2 phosphorylation (Li et al., 2021). Therefore, S1PR3 may regulate BBB damage by activating PI3K/Akt, p38MAPK, and ERK1/2 signaling.

## Discussion

IS is caused by the sudden interruption of arterial blood flow to brain tissue; then, TJs between cerebral microvascular endothelial cells are destroyed, and the expression of the trans-BBB-specific transporter is restricted (Chen et al., 2020). The permeability of the cell side increases, and the influx of immune cells and inflammatory molecules leads to vasogenic edema, hemorrhagic transformation, and increased mortality (Hu et al., 2015). We first analyzed two GEO datasets and became interested in S1PR3, which is closely related to IS. Experimental verification revealed that S1PR3 expression increased after cerebral ischemia and may be involved in the regulation of BBB injury.

S1PR3, a member of the S1P receptor family, plays an essential role in the relevant pathophysiological processes of inflammation, cell proliferation, cell migration, cancer cell invasion and metastasis, I/R, tissue fibrosis, and vascular tone (Fan et al., 2021). Previous studies show that astrocytic S1PR3 is upregulated during the neuroinflammatory response in highly permeable lesions and expressed in patients’ brain metastases. S1PR3 inhibition functionally tightens the blood–tumor barrier (BTB) *in vitro* and *in vivo* (Gril et al., 2018). Therefore, S1PR3 may also be involved in the regulation of BBB injury after tMCAO.

TJ proteins are major structural proteins in both endothelial cells of the BBB and ependymal cells of blood–CSF barriers (Sadowska et al., 2015). Within the CNS vasculature, the BBB is localized at the interface between the blood and cerebral tissue and is composed of endothelial cells connected by an extensive network of complex TJ and adherens junction (AJ) proteins, which influence junction organization at the BBB. Within the BBB, TJ proteins consist primarily of the occludin, claudin, and zonula occludens family of proteins, of which occludin decreases paracellular transport (Bennett et al., 2018). In the process of cerebral ischemia and reperfusion, the expression of occludin along the blood vessel changes from a continuous state to an intermittent state or even disappears, and its decrease has been used to determine the degree of BBB injury (Reinhold and Rittner, 2017). ZO1 is mainly expressed in endothelial and epithelial cells and is an important component involved in the formation of BBB. Several studies have confirmed that a decrease in the expression level and activity of the ZO1 protein will reduce the integrity and stability of the TJ structures between cells (Hue et al., 2015). At the beginning of hypoxia, the ZO1 protein is expressed on the cell membrane, but as the hypoxia time increases, ZO1 gradually migrates into the cytoplasm and then enters the nucleus. Eventually, under hypoxia stimulation, ZO1 migrates from the cell membrane to the cytoplasm, causing the occludin-ZO1-F-actin connection to be broken and the permeability of the BBB to increase. The decrease in the ZO1 expression level indicates the destruction of the BBB (Mark and Davis, 2002). We observed decreased expression of occludin and ZO1 after cerebral ischemia, consistent with previous studies. Inhibition of S1PR3 reduces the permeability of the BBB, cerebral edema, and cerebral infarct volume and increases the expression of occludin and ZO1. The integrity of the BBB is improved. Recently, Xu et al. also found that after intracerebral hemorrhage, inhibiting the S1PR3-CCL2 axis maintains the integrity of the BBB and exerts neuroprotective effects (Xu et al., 2021). Our study revealed an important role for S1PR3 in BBB destruction and brain edema after tMCAO. Excellent results were obtained after CAY-10444 administration, including the amelioration of neurological deficits and improvement in BBB integrity. In-depth studies of S1PR3 as a new target for the development of a therapeutic paradigm and the possibility of discovering new drugs for cerebral infarction will be very important and helpful.

Astrocytes play a crucial role in maintaining the integrity and function of the BBB. They are closely related to BBB permeability through their end feet, which express AQP4. In cerebral ischemia, AQP4 expression is upregulated, blood–brain barrier permeability is increased, and cerebral edema is induced. Therefore, ischemic brain injury is aggravated (Hu et al., 2015). Our study showed that the AQP4 protein level increased after tMCAO, and immunofluorescence staining indicated that the distribution of AQP4 around the blood vessels in mice after cerebral ischemia expanded and was distributed in the extravascular area. This result is consistent with the astrocyte foot processes identified in previous studies. The polarity of the upper AQP4 is missing, and the redistribution matches this change (Steiner et al., 2012; Qiu et al., 2015; Yan et al., 2016). Under normal conditions, AQP4 is coexpressed around blood vessels in mouse brain tissue, and the distribution of AQP4 in the extravascular area increases after ischemia, suggesting the redistribution of AQP4 in mice after cerebral ischemia. After the administration of an S1PR3 inhibitor, the extravascular expression of AQP4 was inhibited, indicating that S1PR3 is involved in the upregulation and redistribution of AQP4.

The degradation of TJPs is mediated by matrix metalloproteinases (MMPs), of which MMP9 is mainly localized in astrocytes (Liu et al., 2020). Previous studies have reported increased expression of ZO1 and occludin after the expression of MMP9 is reduced in a mouse tMCAO model, thereby decreasing the permeability of the BBB (Li et al., 2018). However, we did not observe decreased MMP9 expression after S1PR3 inhibition, indicating that inhibiting S1PR3 attenuates the degradation of ZO1 through a mechanism that does not require reduced MMP9 expression. In fact, TJ proteins are also regulated by PI3K/Akt, MAPK family p38MAPK, ERK1/2, Toll-like receptor 4 (TLR4), and other signal transduction pathways (Liu et al., 2014; Wang et al., 2014; Hue et al., 2015). In the initial KEGG analysis, we also found that S1PR3 may participate in the MAPK signaling pathway, PI3K-Akt signaling pathway, and other pathways during cerebral ischemia and reperfusion injury. Subsequently, AKT phosphorylation decreased after cerebral ischemia and p38 and ERK phosphorylation increased. After inhibiting S1PR3, AKT phosphorylation increased, and p38 and ERK phosphorylation decreased, suggesting that S1PR3 may regulate the breakdown of the BBB through these pathways. The schematic diagram is shown in Figure 1B.

In addition, we verified the results in the GSE22255 peripheral blood sequencing collection and found that S1PR3 was significantly upregulated in patients with IS and was a good IS indicator; therefore, S1PR3 can be used as a marker molecule for predicting cerebral ischemia.

## Conclusion

This article identified that the upregulated S1PR3 was a core gene related to IS by screening differentially expressed genes in the GEO dataset, performing WGCNA, and constructing the PPI network. Then, we performed experimental verification of our results and finally found that S1PR3 participates in the destruction of the integrity of the BBB during cerebral ischemia and reperfusion injury and participates in the regulation of PI3K/Akt, p38MAPK, and ERK1/2 pathways. S1PR3 also represents a potential predictor of cerebral infarction. However, more experiments are needed to verify the role of S1PR3 in cerebral ischemia. These results will help clarify the potential mechanism of BBB destruction after cerebral ischemia at the molecular level, and CAY-10444, a specific inhibitor of S1PR3, is expected to become a clinical drug for cerebral infarction, providing guidance for the pathogenesis, diagnosis, and treatment of cerebral infarction.

## Data Availability

The datasets presented in this study can be found in online repositories. The names of the repository/repositories and accession number(s) can be found in the article/Supplementary Material.

## Supplementary Material

Supplementary Figure S1 PCA analysis shows that the batch effect is better eliminated. **(A)** represents before normalization, **(B)** represents after normalization.

## Abbreviations

S1PR3, sphingosine-1‐phosphate (S1P) receptor 3; MCAO, middle cerebral artery occlusion; GEO, Gene Expression Omnibus; DEGs, differentially expressed genes; WGCNA, weighted gene coexpression network analysis; TJ, tight junction; ZO1, zonula occludens 1; tMCAO, transient MCAO; AQP4, aquaporin 4; IS, ischemic stroke; PPI, protein–protein interaction; I/R, ischemia/reperfusion; MAPK, mitogen-activated protein kinase; PI3K-Akt, phosphatidylinositol-3 kinase/serine-threonine kinase; FDA, Food and Drug Administration; BBB, blood–brain barrier; ERK, Ras/extracellular signal-regulated kinase; PBMCs, peripheral blood mononuclear cells; TOM, topological overlap matrix; GS, gene significance; MM, module member; GO, Gene Ontology; BPs, biological processes; MFs, molecular functions; CCs, cellular components; KEGG, Kyoto Encyclopedia of Genes and Genomes; PVDF, polyvinylidene fluoride; BTB, blood–tumor barrier; TLR4, Toll-like receptor 4; TNF, tumor necrosis factor; EB, Evans Blue.
