# Supplementary figures and images for: S1PR3, as a Core Protein Related to Ischemic Stroke, is Involved in the Regulation of Blood–Brain Barrier Damage
Source: Front Pharmacol. 2022 May 24;13:834948. doi: 10.3389/fphar.2022.834948 (PMC9173650; doi:10.3389/fphar.2022.834948)

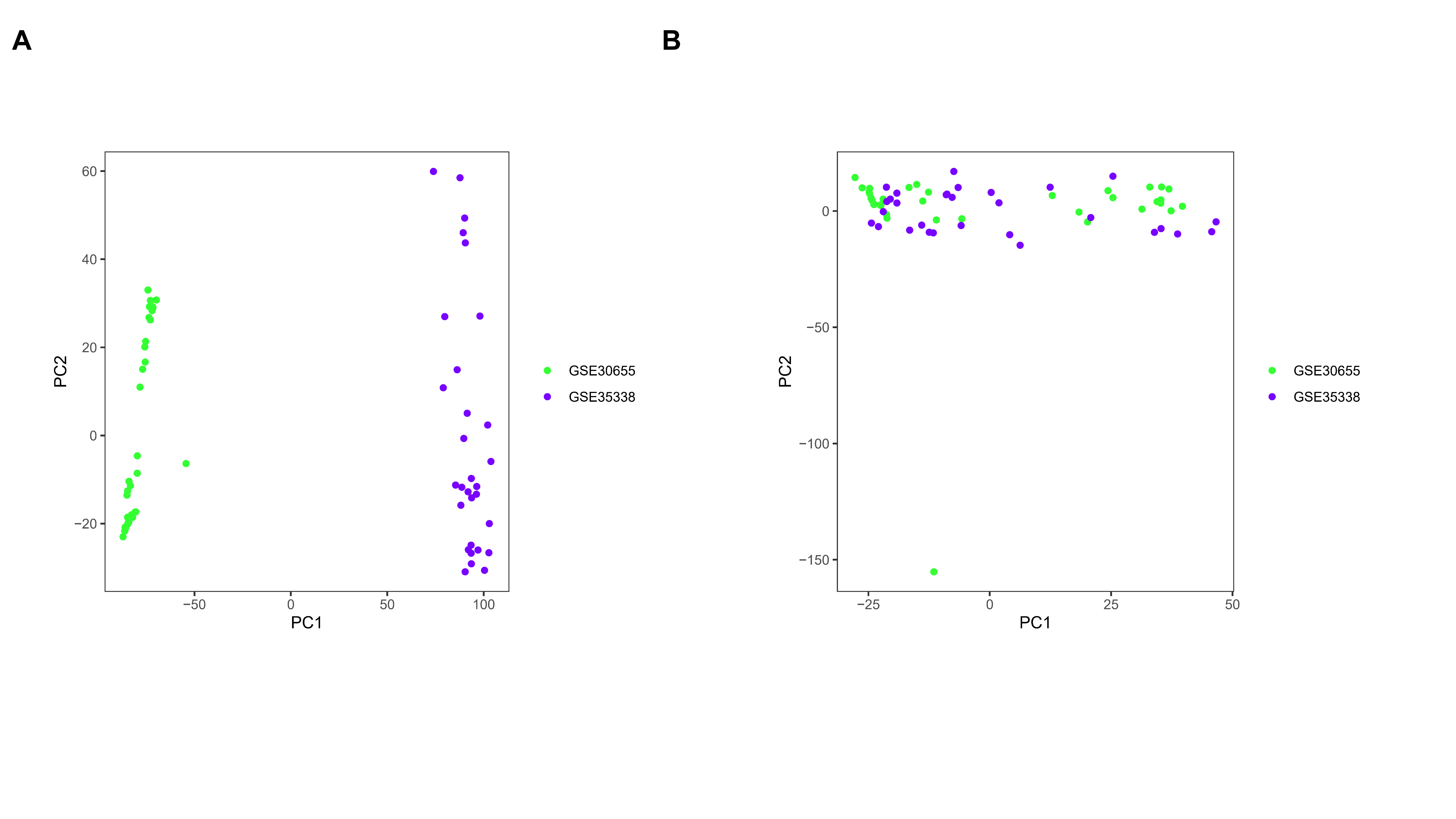

Supplement: Supplementary file 1 [file Figure1.tif]
